# Post treatment NLR is a predictor of response to immune checkpoint inhibitor therapy in patients with esophageal squamous cell carcinoma

**DOI:** 10.1186/s12935-021-02072-x

**Published:** 2021-07-07

**Authors:** Xianbin Wu, Runkun Han, Yanping Zhong, Nuoqing Weng, Ao Zhang

**Affiliations:** 1 Department of Gastrointestinal Surgery, The Eighth Affiliated Hospital, Sun Yat-sen University, Shenzhen, 518033 China; 2 Department of Laboratory Medicine, State Key Laboratory of Oncology in South China, Collaborative Innovation Center for Cancer Medicine, Sun Yat-sen University Cancer Center, Guangzhou, 510060 China; 3 Department of Health Management, The Eighth Affiliated Hospital, Sun Yat-sen University, Shenzhen, China

**Keywords:** Esophageal squamous cell carcinoma, Anti-PD-1 treatment, Progression-free survival, Neutrophil-to-lymphocyte ratio

## Abstract

**Background:**

In view of the fact that peripheral blood parameters have been reported as predictors of immunotherapy to various cancers, this study aimed to determine the predictors of response to anti-programmed death-1 (anti-PD-1) therapy in patients with esophageal squamous cell carcinoma (ESCC) from peripheral blood parameters.

**Methods:**

A retrospective analysis was conducted to investigate the predictive value of peripheral blood parameters including neutrophil-to-lymphocyte ratio (NLR), platelet-to-lymphocyte ratio (PLR), monocyte-to-lymphocyte ratio (MLR) and systemic immune-inflammation index (SII) in the response to anti-PD-1 antibody treatment. 119 ESCC patients receiving combined treatment including anti-PD-1 antibody were enrolled in this study.

**Results:**

The median progression-free survival (PFS) of all ESCC patients was 3.73 months. PFS rate in ESCC patients with low NLR at 6 weeks post treatment was higher than patients with high NLR (HR = 2.097, 95% CI 0.996–4.417, P = 0.027). However, PFS rate in ESCC patients with low NLR at baseline (HR = 1.060, 95% CI 0.524–2.146, P = 0.869) or 3 weeks post treatment (HR = 1.293, 95% CI 0.628–2.663, P = 0.459) was comparable with high NLR. And no statistically different was found in PFS rate between low PLR and high PLR at baseline (HR = 0.786, 95% CI 0.389–1.589, P = 0.469), 3 weeks post treatment (HR = 0.767, 95% CI 0.379–1.552, P = 0.452) or 6 weeks post treatment (HR = 1.272, 95% CI 0.624–2.594, P = 0.488) in ESCC patients. PFS rate was also comparable between low MLR and high MLR at baseline (HR = 0.826, 95% CI 0.408–1.670, P = 0.587), 3 weeks post treatment (HR = 1.209, 95% CI 0.590–2.475, P = 0.580) or 6 weeks post treatment (HR = 1.199, 95% CI 0.586–2.454, P = 0.596). PFS rate was similar between patients with low SII and high SII at baseline (HR = 1.120, 95% CI 0.554–2.264, P = 0.749), 3 weeks post treatment (HR = 1.022, 95% CI 0.500–2.089, P = 0.951) and 6 weeks post treatment (HR = 1.759, 95% CI 0.851–3.635, P = 0.097).

**Conclusions:**

NLR at 6 weeks post treatment is a predictor of the response to anti-PD-1 treatment in patients with ESCC.

## Introduction

Esophageal cancer (EC) with a poor overall 5-year survival rate ranging from 15 to 25% ranks the eighth most commonly diagnosed cancer worldwide, while the sixth most common cancer in China [1]. Esophageal squamous cell carcinoma (ESCC) predominantly found in Asia, Africa, and South America, and esophageal adenocarcinoma (EAC) predominant in North America and Europe are the two main subtypes of EC [2]. As a highly aggressive squamous cell carcinoma, ESCC occupies the main subtype of EC in China because of special living habits [3]. Due to the lack of obvious symptoms of ESCC at early stage, patients are often diagnosed at advanced stage and lose the opportunity for surgery, as a result of which, chemotherapy and other treatments appear to be particularly important [4]. Unfortunately, the effect of chemotherapy on patients with advanced ESCC is not ideal. Up to now, there is no effective targeted therapy for EC patients [5, 6]. Hence, the high recurrence and metastasis rate of patients, and the low 5-year survival rate make the therapy of ESCC still a big problem.

In recent years, patients suffered diverse types of cancer have had benefits from immune checkpoint inhibitors (ICIs) therapies, which were principally represented by programmed death 1/programmed death ligand 1 (PD-1/PD-L1) inhibitors [7, 8]. Inspired by such good news, many clinical trials of ICIs were constructed in patients with advanced ESCC. For example, a randomized, open-label, phase 3 study named ESCORT found that patients with advanced or metastatic esophageal squamous cell carcinoma who had previously failed first-line chemotherapy and received camrelizumab alone significantly extended survival when compared with the investigator-selected chemotherapy [9]. These studies showed quite a remarkable clinical benefit from anti-PD-1/PD-L1 antibody in advanced ESCC patients. Nevertheless, there is no reliable predictor of anti-PD-1 treatment efficacy in patients with ESCC. Therefore, there is an urgent need to identify an effective indicator for predicting survival benefits from anti-PD-1 treatment in patients with ESCC.

It has been reported that cancer-related inflammation is significantly associated with tumor progression and survival in patients with different types of cancer [10]. Alteration of peripheral blood biomarkers are capable of representing the systemic inflammation in patients such as neutrophil-to-lymphocyte ratio (NLR), platelet-to-lymphocyte ratio (PLR) and systemic immune-inflammation index (SII) which was defined as follows: SII = platelet × neutrophil / lymphocyte. As the reports before, more and more peripheral blood biomarkers were found to be correlated with the outcomes for ICIs treatments in diverse types of cancer. For instance, a composite model of post-treatment NLR and PLR was recognized in 103 HCC patients with anti-PD-1 treatment to predict therapeutic qualities. A combination of high NLR and PLR were associated with high risk of death in this study, indicating that inflammatory cell ratios at the post-treatment in patients with hepatocellular carcinoma (HCC) played a strong predictive role in response to anti-PD-1 treatment [11]. Furthermore, a multicenter retrospective study was reported that a combined baseline serum biomarker including derived NLR (dNLR) which was defined as follows: dNLR = neutrophil count / (white blood cell count—neutrophil count) and lactate dehydrogenase (LDH) was able to be a predictor of anti-PD-1 treatment efficacy in 466 patients with non-small cell lung cancer (NSCLC). The pretreatment SII also might be a useful indicator for predicting survival in NSCLC patients after anti-PD-1 antibody treatment [12].

Up to now, there is no study on evaluating the role of peripheral blood parameters in ESCC patients with anti-PD-1/PD-L1 antibody treatment. Hence, we conducted a retrospective study involving 119 ESCC patients with PD-1 inhibitor therapy to evaluate the prognostic value of peripheral blood biomarkers. In this study, we aim to make up a reliable, convenient and minimally invasive prognostic indicator for predicting the response of anti-PD-1 combined therapy in ESCC patients.

## Patients and methods

### Patients and tumor-free people

119 patients with ESCC who received anti-PD-1 inhibitor treatment from December 2018 to September 2020 in Sun Yat-sen University Cancer Center, Guangzhou were included in this retrospective study. All patients were firstly treated with PD-1 inhibitor at least 3 weeks and measured complete blood counts at the beginning of treatment (within 3 days before the first treatment), 3 weeks later and 6 weeks later. The response to PD-1 inhibitor treatment in ESCC patients was firstly evaluated at 8–12 weeks and updated continuously after treatment. The clinical characteristics of patients with ESCC, such as age, gender, alcohol, smoking, metastasis, recurrence, TNM classification and so on were collected. And 818 cases of tumor-free people in Cancer Prevention Health Center of Sun Yat-sen University Cancer Center, Guangzhou during April 2021 were also collected.

### Assessment

According to RECIST (solid tumor response assessment criteria) v1.1, radiological examinations were performed to evaluate the effect of immunotherapy at 8–12 weeks. The response of patients to PD-1 inhibitor was including complete remission (CR), partial remission (PR), stable disease (SD) and progressive disease (PD). The time from the start date of PD-1 inhibitor treatment to the date of disease progression or death was calculated for each patient as PFS. Clinical response was defined as CR, PR and SD, while PD was defined as non-clinical response.

### Peripheral blood parameters

The peripheral blood test results including neutrophil (NE), lymphocyte (LY), monocyte (MO) and platelet (PLT) were collected respectively. In order to evaluate the systemic inflammation of patients with ESCC, NLR, MLR, PLR and SII were calculated according to the following rules: NLR was calculated as NE divided by the LY, and PLR was calculated as PLT divided by the LY; MLR was defined as the ratio of MO to LY; And SII was calculated as PLT multiplied by NE and then divided by LY. The threshold values of the above parameters were the median of themselves, respectively. ΔNLR meant the difference value of post-treatment NLR and baseline NLR.

### Statistical analysis

Categorical variables were summarized as frequencies and percentages. The Kaplan–Meier survival method was used to evaluate the probability of PFS, and Log Rank test was used to estimate significance of the differences between groups. Statistical analysis of composition ratio of patients with low NLR and with high NLR at 6 weeks post treatment was performed using Pearson's chi-square test and Fisher exact test. The difference of distribution of NLR between ESCC patients and tumor-free people was analyzed by Pearson's chi-square test. Statistical analysis of change of NLR in patients of response and non-response groups was also performed by Pearson's chi-square test and Fisher exact test. P < 0.05 was considered statistically significant. Graphpad Prism 8.0 (GraphPad Software, La Jolla, CA, USA) and SPSS 23.0 (IBM Corp., Armonk, NY, USA) were used for statistical analysis.

## Result

### Clinical characteristics of ESCC patients

Clinical characteristics of ESCC patients were summarized in Table 1. A total of 119 patients including 102 males and 17 females were involved in this study. Patients ranged in age from 42 years old to 78 years old and the median age of ESCC patients in this study was 61 years old. There were 75 (63.0%) smokers and 44 (37.0%) non-smokers. While 63 (52.9%) patients were in the habit of drinking, and 56 (47.1%) patients were not in the habit. There were 54 (45.4%) patients with tumor metastasis and 65 (54.6%) patients without tumor metastasis. In our study, most of patients were diagnosed at advanced TNM stage. 26 cases of (21.8%) ESCC patients suffered tumor recurrence, while 93 (78.2%) cases of patients did not recurrence (Table 1). All patients were received combined treatment including surgery, radiotherapy or chemotherapy. And all patients were treated with anti-PD-1 antibody, and the varieties of the anti-PD-1 antibody were as follow: camrelizumab, nivolumab, pembrolizumab, toripalimab and sintilimab. The follow-up time was ended on October 1st 2020, and the median PFS was 3.73 months.

**Table 1:** Clinical characteristics of ESCC patients

Characteristics (n = 119)	N(%)
Age	
Median	61
Range	42–78
< 60	51(42.9)
≥ 60	68(57.1)
Sex	
Female	17(14.3)
Male	102(85.7)
Smoking	
Yes	75(63.0)
No	44(37.0)
Alchohol	
Yes	63(52.9)
No	56(47.1)
Metastasis	
Yes	54(45.4)
No	65(54.6)
TNM stage	
I–II	8(6.70)
III–IV	111(93.3)
Recurrence	
Yes	26(21.8)
No	93(78.2)

### Association between response to anti-PD-1 treatment and NLR at baseline, at 3 weeks post treatment and at 6 weeks post treatment

**Fig. 1:**
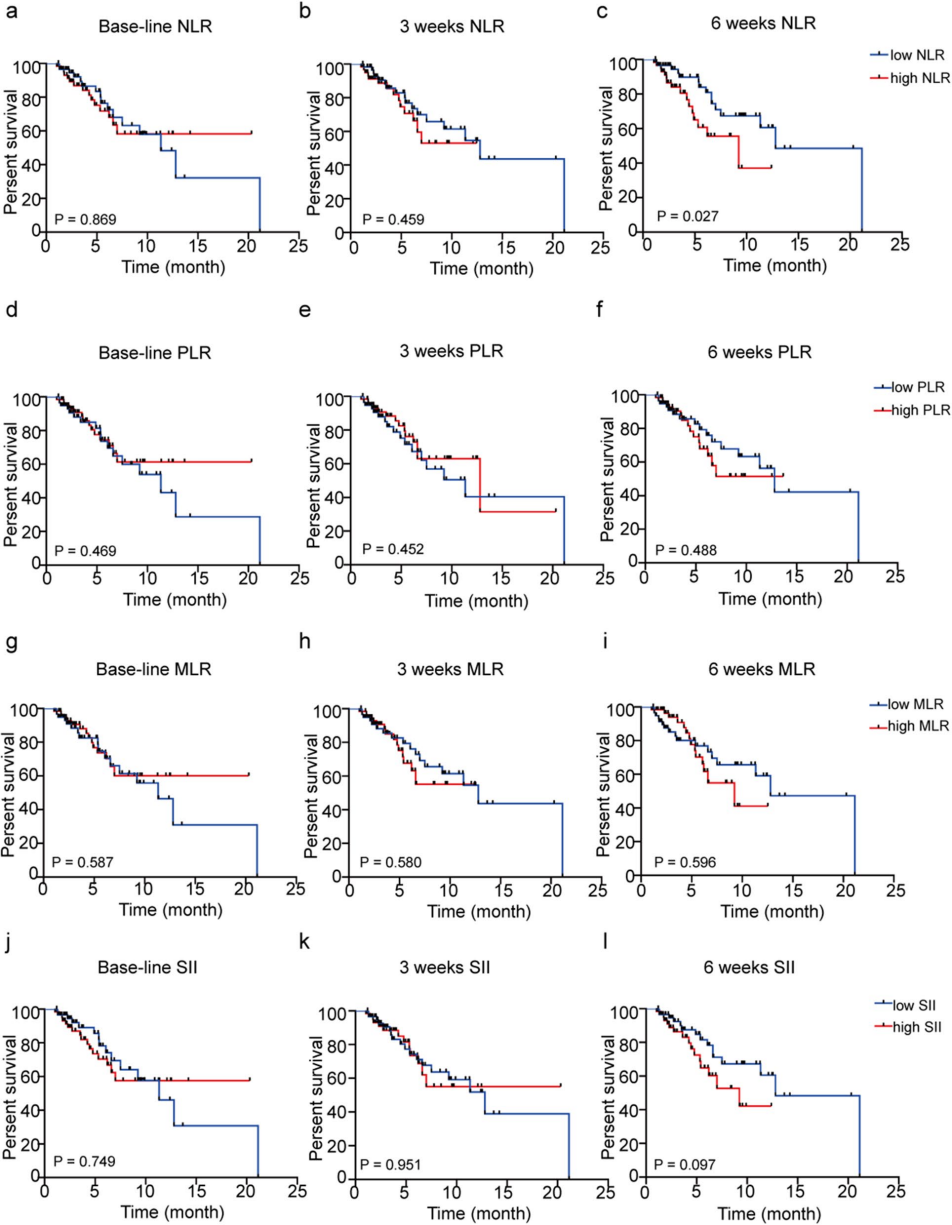
Association between response to anti-PD-1 treatment and NLR, PLR, MLR, SII at baseline, at 3 weeks post treatment and at 6 weeks post treatment. The patients were defined as high or low groups by NLR, PLR, MLR or SII level and then analyzed with Kaplan–Meier survival curves. **a** Progression-free survival (PFS) curves of patients with baseline low NLR and high NLR. **b** Progression-free survival (PFS) curves of patients with low NLR and high NLR at 3 weeks. **c** Progression-free survival (PFS) curves of patients with low NLR and high NLR at 6 weeks. **d** Progression-free survival (PFS) curves of patients with baseline low PLR and high PLR. **e** Progression-free survival (PFS) curves of patients with low PLR and high PLR at 3 weeks. **f** Progression-free survival (PFS) curves of patients with low PLR and high PLR at 6 weeks. **g** Progression-free survival (PFS) curves of patients with baseline low MLR and high MLR. **h** Progression-free survival (PFS) curves of patients with low MLR and high MLR at 3 weeks. **i** Progression-free survival (PFS) curves of patients with low MLR and high MLR at 6 weeks. **j** Progression-free survival (PFS) curves of patients with baseline low SII and high SII. **k** Progression-free survival (PFS) curves of patients with low SII and high SII at 3 weeks. **l** Progression-free survival (PFS) curves of patients with low SII and high SII at 6 weeks

The ESCC patients were divided into low NLR and high NLR groups according to baseline NLR, with the threshold value at 3.23, which was the median of baseline NLR in our cohort. The difference of PFS between two groups was not statistically significant (HR = 1.060, 95% CI 0.524–2.146, P = 0.869; Fig. 1a). Then the patients were divided into two groups according to 3 weeks NLR with the threshold value at 3.34, which was the median of 3 weeks NLR and 6 weeks NLR with the threshold value at 3.69, which was the median of 6 weeks NLR, respectively. The difference of PFS between low NLR and high NLR groups according to 3 weeks NLR was also not statistically significant (HR = 1.293, 95% CI 0.628–2.663, P = 0.459; Fig. 1b). While patients were then separated into two groups according to NLR at 6 weeks post treatment, median PFS was significantly longer in patients with low NLR than patients with high NLR (12.80 vs. 9.23 months, respectively; HR = 2.097, 95% CI 0.996–4.417, P = 0.027; Fig. 1c). Additionally, the clinical characteristics were comparable between low NLR and high NLR groups at 6 weeks post treatment (Table 2).

**Table 2:** The relationships of NLR at 6 weeks post treatment and clinical characteristics of ESCC patients

Characteristics (n = 119)	Low NLR	High NLR	P value
Age			0.397
< 60	28	23	
≥ 60	32	36	
Sex			0.299
Female	10	6	
Male	50	53	
Smoking			0.757
Yes	37	38	
No	23	21	
Alchohol			0.650
Yes	33	30	
No	27	29	
Metastasis			0.079
Yes	32	22	
No	28	37	
TNM stage			1.000
I–II	4	4	
III–IV	56	55	
Recurrence			0.166
Yes	16	10	
No	42	49	

χ^2^ test or Fisher’s exact test were conducted

### Association between response to anti-PD-1 treatment and other peripheral blood parameters at baseline, at 3 weeks post treatment and at 6 weeks post treatment

According to the threshold value which was the median of baseline PLR, 3 weeks PLR or 6 weeks PLR, patients were divided into low PLR and high PLR groups, respectively. PFS rate of patients was comparable between the low PLR and high PLR groups at the beginning of treatment (HR = 0.786, 95% CI 0.389–1.589, P = 0.469; Fig. 1d), 3 weeks post treatment (HR = 0.767, 95% CI 0.379–1.552, P = 0.452; Fig. 1e) and 6 weeks post treatment (HR = 1.272, 95% CI 0.624–2.594, P = 0.488; Fig. 1f). While PFS rate of patients were also comparable between low MLR and high MLR groups at the beginning of treatment (HR = 0.826, 95% CI 0.408–1.670, P = 0.587; Fig. 1g), 3 weeks post treatment (HR = 1.209, 95% CI 0.590–2.475, P = 0.580; Fig. 1h) and 6 weeks post treatment (HR = 1.199, 95% CI 0.586–2.454, P = 0.596; Fig. 1i). The 119 patients were dichotomized using a threshold value of the median of baseline SII, 3 weeks SII or 6 weeks SII, respectively. At baseline, PFS rate was not significantly different between low SII group and high SII group (HR = 1.120, 95% CI 0.554–2.264, P = 0.749; Fig. 1j). At 3 weeks post treatment, there was no significant difference of PFS rate between low SII group and high SII group (HR = 1.022, 95% CI 0.500–2.089, P = 0.951; Fig. 1k). While at 6 weeks post treatment, the PFS rate was comparable between low SII group and high SII group (HR = 1.759, 95% CI 0.851–3.635, P = 0.097; Fig. 1l).

### Analysis of NLR in general population

Since NLR was significantly important in predicting anti-PD-1 antibody treatment in patients with ESCC, we collected 818 cases of people whose median age was 53 years old and ranging from 44 years old to 78 years old including 430 males and 388 females without any type of tumors from Cancer Prevention Health Center to evaluate difference of NLR between ESCC patients and tumor-free people (Tables 3, 4 and 5). Then we selected 238 cases of tumor-free people, whose median age was 59 years old and age range was from 44 years old to 78 years old. Meanwhile, the ratio of males to females in 238 cases of tumor-free people was the same as the ratio in ESCC patients. The median of NLR in tumor-free people was 1.66, while the median of NLR at baseline in ESCC patients was 3.23, suggesting an increasing trend of NLR in patients with ESCC when compared with tumor-free people. Furthermore, we analyzed NLR of 818 cases of tumor-free people and found that the median of NLR in 818 people was 1.61, which was similar with the median NLR in 238 cases of tumor-free people. The median NLR of males in tumor-free people was 1.64, while median NLR of females was 1.58. After that, we evaluated whether age was able to influence NLR level in tumor-free people and found that the median of NLR was 1.64 in people with age less than 60 years old and median of NLR was 1.51 in people with age greater than or equal to 60 years old. Lastly, we analyzed the difference of distribution of NLR greater than 3.23, which was the median NLR in ESCC patients between tumor-free people and ESCC patients and found that there were 795 cases of tumor-free people with NLR less than 3.23 and only 23 cases of tumor-free people with NLR greater than or equal to 3.23 (p < 0.05), further demonstrating that people with tumors were under hyperinflammatory state, which was consistent with previous study.

**Table 3:** Essential characteristics of tumor-free people

Characteristics	N(%)
238 cases	
Age	
Median	59
Range	44–78
Sex	
Male	204(85.7)
Female	34(14.3)
818 cases	
Age	
Median	53
Range	44–78
Sex	
Male	430(52.6)
Female	388(47.4)

**Table 4:** Analysis of NLR in general population

Group	NLR
ESCC patients	3.23
238 cases	1.66
818 cases	
All	1.61
Age	
< 60	1.64
≥ 60	1.51
Sex	
Male	1.64
Female	1.58

**Table 5:** Analysis of the difference of NLR between tumor-free people and ESCC patients

Group	NLR level		
	< 3.23	≥ 3.23	Total
ESCC patients	60	59	119
Tumor-free people	795	14	818
Total	855	73	937
P value	< 0.05		

### Analysis of PLR, MLR and SII in general population

NLR level at baseline was increased in patients with ESCC when compared with tumor-free people, therefore we calculated PLR, MLR and SII in general population to assess the influence of tumor on alteration of PLR, MLR and SII (Table 6). Then we calculated PLR of 818 cases of tumor-free people and found that median of PLR in tumor-free people was 117.01, while median of PLR at baseline in ESCC patients was 174.72. The median of MLR in tumor-free people was 0.30, meanwhlie the median of MLR at baseline in ESCC patients was 0.21. And the median of SII in tumor-free people was 376.76, while the median of SII at baseline in ESCC patients was 829.37. The above results displayed that PLR and SII showed an increase in ESCC patients when compared with tumor-free people.

**Table 6:** Analysis of PLR, MLR and SII in general population

Parameter	ESCC patients	Tumor-free people
PLR	174.72	117.01
MLR	0.21	0.30
SII	829.37	376.76

### Trend analysis of NLR during treatment

**Fig. 2:**
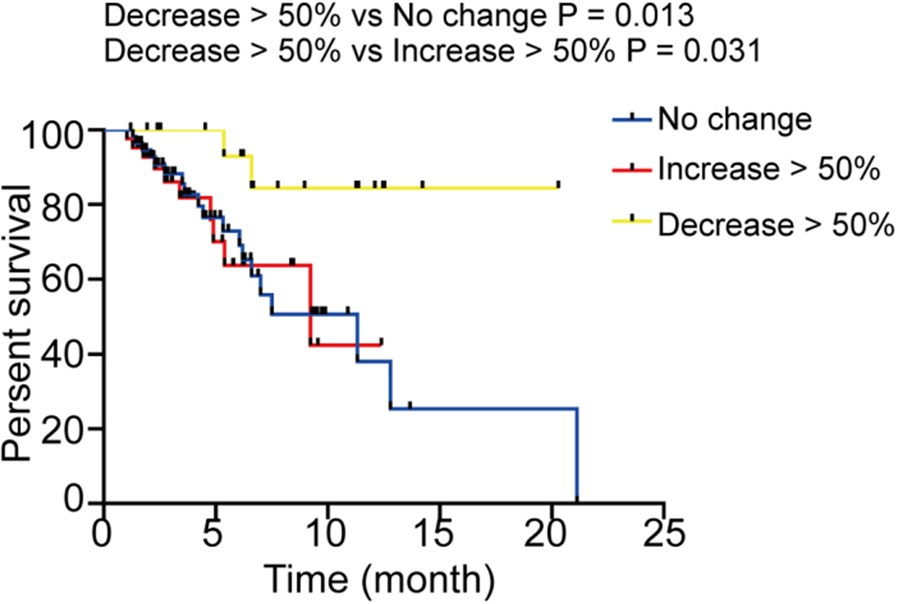
Association between response to anti-PD-1 treatment and 6 weeks NLR decrease > 50%, increase > 50% and no change in ESCC patients. The patients were defined as decrease > 50%, increase > 50% and no change groups by the difference between baseline NLR and 6 weeks NLR and then analyzed with Kaplan–Meier survival curves

The above analysis demonstrated that high NLR at 6 weeks post treatment but not at baseline or at 3 weeks was related to shorter PFS in ESCC patients and NLR was significantly elevated in ESCC patients when compared with tumor-free people, thus we estimated whether the change of NLR between baseline and 6 weeks post treatment was responsible for the response to anti-PD-1 treatment. Patients were divided into response and non-response groups according to the clinical response or not. Decrease in NLR was shown in 36/88 (40.9%) patients in the response group while 14/31 (45.2%) in the non-response group, and the number of patients with decrease in NLR was comparable in the response group and non-response group (P = 0.679; Table 7). However, if we set a threshold value at 50% of the percentage change of NLR, there were 16/88 (18.2%) patients with decrease in NLR in the response group and 2/31 (6.45%) patients with decrease in NLR in the non-response group, which was not statistically significant although the number of patients in two groups varied (P = 0.187; Table 8). Then the patients were divided into three groups including > 50% decrease group, no change group and > 50% increase group according to the percentage change of NLR at the threshold value of 50%. PFS rate in > 50% decrease group was significantly different with no change group (HR = 0.199, 95% CI 0.080–0.488, P = 0.013; Fig. 2) or > 50% increase group (HR = 0.228, 95% CI 0.073–0.710, P = 0.031; Fig. 2) which suggested that decrease in NLR was an important factor after anti-PD-1 antibody treatment.

**Table 7:** Changes of NLR in patients of response and non-response groups

Group	Trend		
	Decrease	Increase	Total
Response	36	52	88
Non-response	1714	1417	31
Total	5350	6669	119
P value	0.679

**Table 8:** Analysis of over 50% percentage change of NLR in patients of response and non-response groups

Group	Trend			
	Decrease > 50%	Increase > 50%	No change	Total
Response	16	32	40	88
Non-response	2	10	19	31
Total	18	42	59	119
P value	0.187

## Discussion

Anti-PD-1/PD-L1 therapies have shown promising therapeutic effect in patients with metastatic esophageal cancer in many clinical trials [13, 14]. Although anti-PD-1/PD-L1 antibody have been applied in patients with ESCC, there are still no reliable biomarkers to predict the effect of anti-PD-1/PD-L1 antibody treatment. Therefore, it is urgent for us to find out effective biomarkers to estimate the efficacy of anti-PD-1/PD-L1 treatment in patients with ESCC. In previous study, peripheral blood parameters can reflect the system state of human bodies [15–17]. NLR and other hematologic parameters have become biomarkers to predict the overall survival (OS) and anti-PD-1 / PD-L1 treatment effect of diverse types of cancer [18, 19]. PLR, MLR and SII were also reported to be potential biomarkers for predicting OS and the response to anti-PD-1/PD-L1 antibody treatment in different types of cancer [20–23]. For example, NLR, MLR and PLR have clinical utility for predicting survival in patients with advanced gastric cancer and colorectal cancer [23]. While high levels of SII (≥ 720), NLR (≥ 4.3) and cytokine IFN-inducible protein-10 (IP-10; ≥ 45 pg/ml) indicated worse OS in advanced biliary tract cancer [24]. Neutrophil-to-lymphocyte ratio at 6 weeks post treatment in patients with advanced NSCLC was able to predict PFS after anti-PD-1/PD-L1 treatment [25]. Moreover, high baseline NLR and high baseline PLR were both associated with worse immunotherapy efficacy regardless of cancer type, especially in metastatic melanoma and NSCLC [26].

Therefore, we hypothesized that the above parameters which were able to reflect the inflammation and adaptive immune response in diverse malignant tumors also could be promising predictors of the response to anti-PD-1 antibody treatment in ESCC patients [23, 27–30]. In this study, we evaluated whether NLR, PLR, MLR or SII was able to be a biomarker for predicting response to anti-PD-1 antibody treatment in ESCC patients. It was the first study of estimating the association between peripheral blood parameters and the response to anti-PD-1 antibody in over 100 cases of ESCC patients. Based on our data, we found that NLR at 6 weeks post treatment was associated with the response to anti-PD-1 antibody treatment and low NLR after anti-PD-1 treatment seemed to be a favorable factor in the therapeutic effect on anti-PD-1 treatment in ESCC patients, while NLR at baseline or 3 weeks post treatment might not influence PFS rate in ESCC patients. PFS rate in patients with decrease NLR at 6 weeks post treatment compared with baseline NLR were higher than patients with no change NLR or increase NLR. In previous study, patients were divided into low NLR and high NLR groups with the threshold value set as 5, and patients with low NLR had longer OS than high NLR in many types of cancer [29, 31–34]. However, in our study, only a little percentage of NLR were greater than 5 in ESCC patients and PFS rate in patients with NLR > 5 was comparable with patients with NLR ≤ 5, as a result of which, we set a new cutoff value at the median of NLR, and then divided patients into low NLR and high NLR groups which was reasonable according to previous studies [32, 35]. And we also explored the role of PLR, MLR and SII in predicting the response to anti-PD-1 antibody in ESCC patients in our study. Unfortunately, PLR, MLR and SII were not associated with the response to the anti-PD-1 treatment in ESCC patients, as a result of which, it was assuming that NLR was a vital factor reflecting the system inflammation response after anti-PD-1 therapy. Neutrophil and lymphocyte were important components of tumor immune microenvironment and played a role in tumor-associated immunity [36–38], but the mechanism of the significance of peripheral blood NLR which representing systemic inflammation in tumor-associated immunity was still unclear. In our study, we also evaluated the NLR level of the tumor free population and found that the NLR level of ESCC patients had a significantly higher trend than that of the tumor free population. Age and gender were not the important factors affecting NLR level in our study, which is consistent with the previous reports that tumor patients are in a hyperinflammatory state [39, 40]. Tumor patients have higher NLR levels because NLR reflects systemic inflammation [41]. Anti-PD-1 antibodies aim at PD-1 antigen expressed on membrane of CD8 + T cells and influence systemic inflammation in patients, accompanied by the change of NLR [42–44]. Meanwhile, NLR at 6 weeks after treatment may be an appropriate time to evaluate the correlation between NLR and anti-PD-1 antibody treatment response in ESCC patients according to previous research [25, 32, 43].

There are still some limitations existing in our study. Firstly, this study was only a single center retrospective analysis with small sample size, meanwhile, some bias and confounding factors are inescapable. For instance, patients with low SII at 6 weeks post treatment reached marginal association with PFS, which might be caused by small sample size. Secondly, overall survival was not available for our analysis considering the follow-up time. Thirdly, peripheral blood parameters were influenced by many other factors, which could not be excluded completely. Last but not the least, the immune mechanism of this phenomenon was still unclear and needed further exploration. Nevertheless, our study has provided a simple, convenient and noninvasive biomarker to predict the response to anti-PD-1 antibody therapy in ESCC patients which may be helpful to develop individualized treatment.

## Conclusions

In this study, we identified that NLR at 6 weeks after anti-PD-1 treatment was able to predict the response to anti-PD-1 antibody in patients with ESCC, and a decrease in NLR after treatment predicted a better treatment response to anti-PD-1 therapy.

## Data Availability

The datasets used and/or analyzed during the current study are available from the corresponding author on reasonable request.

## Abbreviations

ESCC: Esophageal squamous cell carcinoma
PD-1: Programmed death 1
PD-L1: Programmed death ligand 1
WBC: White blood cell
NLR: Neutrophil-to-lymphocyte ratio
PLR: Platelet-to-lymphocyte ratio
MLR: Monocyte-to-lymphocyte ratio
SII: Systemic immune-inflammation index
LDH: Lactic dehydrogenase
PFS: Progression-free survival
OS: Overall survival
CR: Complete remission
PR: Partial remission
SD: Stable disease
PD: Progressive disease
HR: Hazard ratio
95% CI: 95% Confidence interval
