# Correction to: Post treatment NLR is a predictor of response to immune checkpoint inhibitor therapy in patients with esophageal squamous cell carcinoma

**DOI:** 10.1186/s12935-021-02401-0

**Published:** 2021-12-20

**Authors:** Xiaobin Wu, Runkun Han, Yanping Zhong, Nuoqing Weng, Ao Zhang

**Affiliations:** 1 Department of Gastrointestinal Surgery, The Eighth Affiliated Hospital, Sun Yat-sen University, Shenzhen, 518033 China; 2 Department of Laboratory Medicine, State Key Laboratory of Oncology in South China, Collaborative Innovation Center for Cancer Medicine, Sun Yat-sen University Cancer Center, Guangzhou, 510060 China; 3 Department of Health Management, The Eighth Affiliated Hospital, Sun Yat-sen University, Shenzhen, China

## Correction to: Cancer Cell Int (2021) 21:356 10.1186/s12935-021-02072-x

In this article [1], the author name “Xiaobin Wu” was incorrectly written as “Xianbin Wu”. This has been corrected in this correction.

In addition, there was a printing mistake in Table 7. The correct information of 1714 is 14, 1417 is 17, 5350 is 50 and 6669 is 69. The corrected Table 7 is given below.

**Table 7:** Changes of NLR in patients of response and non-response groups

Group	Trend		
	Decrease	Increase	Total
Response	36	52	88
Non-response	14	17	31
Total	50	69	119
P value	0.679		
